# A qualitative formative evaluation of a patient‐centred patient safety intervention delivered in collaboration with hospital volunteers

**DOI:** 10.1111/hex.12560

**Published:** 2017-06-15

**Authors:** Gemma Louch, Jane O'Hara, Mohammed A. Mohammed

**Affiliations:** ^1^ Bradford Institute for Health Research Bradford Royal Infirmary Bradford UK; ^2^ Leeds Institute of Medical Education University of Leeds Leeds UK; ^3^ Faculty of Health Studies University of Bradford Bradford UK

**Keywords:** evaluation, improvement science, patient feedback, patient involvement, patient safety, volunteers

## Abstract

**Background:**

Evidence suggests that patients can meaningfully feed back to healthcare providers about the safety of their care. The PRASE (Patient Reporting and Action for a Safe Environment) intervention provides a way to systematically collect feedback from patients to support service improvement. The intervention is being implemented in acute care settings with patient feedback collected by hospital volunteers for the first time.

**Objective:**

To undertake a formative evaluation which explores the feasibility and acceptability of the PRASE intervention delivered in collaboration with hospital volunteers from the perspectives of key stakeholders.

**Design:**

A qualitative evaluation design was adopted across two acute NHS trusts in the UK between July 2014 and November 2015. We conducted five focus groups with hospital volunteers (n=15), voluntary services and patient experience staff (n=3) and semi‐structured interviews with ward staff (n=5). Data were interpreted using framework analysis.

**Results:**

All stakeholders were positive about the PRASE intervention as a way to support service improvement, and the benefits of involving volunteers. Volunteers felt adequate training and support would be essential for retention. Staff concentrated on the infrastructure needed for implementation and raised concerns around sustainability. Findings were fed back to the implementation team to support revisions to the intervention moving into the subsequent summative evaluation phase.

**Conclusion:**

Although there are concerns regarding sustainability in practice, the PRASE intervention delivered in collaboration with hospital volunteers is a promising approach to collect patient feedback for service improvement.

## 1 INTRODUCTION

Patients are increasingly being regarded as partners in their care and evidence is building that patients can willingly and meaningfully feed back on the safety of their care to healthcare providers.^1^, ^2^ However, until recently there has been no evidence‐based approach for the systematic collection of such feedback from patients in hospital ward settings. The PRASE intervention was developed and codesigned with staff and patients to address this need. The intervention supplies healthcare providers with theory and evidence‐based measurement tools for the routine collection of feedback around the safety of care from patients, alongside a framework for staff to interpret and act on that feedback.^1^, ^3^, ^4^, ^5^, ^6^, ^7^

**Figure 1:**
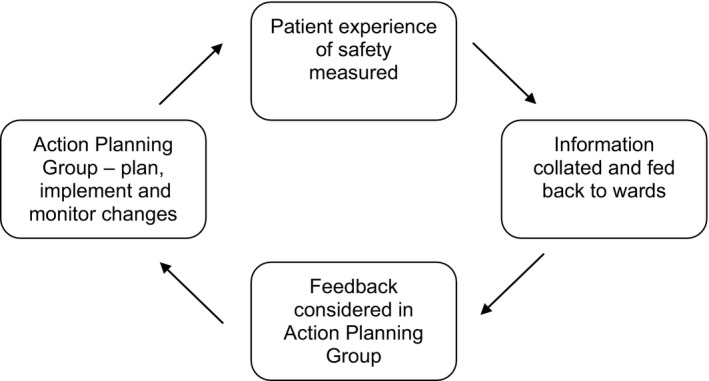
The PRASE intervention cycle

The efficacy of the PRASE intervention in achieving patient safety improvements over a 12‐month period has previously been explored in a randomized controlled trial^5^ with an embedded qualitative process evaluation.^8^ The intervention comprises two measurement tools: first, the Patient Measure of Safety (PMOS),^3^, ^4^ which is a questionnaire based on the content of the Yorkshire Contributory Factors Framework;^9^ second, the Patient Incident Reporting Tool (PIRT),^1^, ^6^ which enables patients to report detailed safety concerns and/or positive experiences. The anonymous feedback collected using these tools is presented to ward staff in the form of a feedback report. An iterative action planning cycle follows whereby ward staff consider the feedback in an action planning meeting and target improvements based on problematic areas. The three key stages of the PRASE intervention process are (i) measurement, (ii) feedback and (iii) action planning and change (see Figure 1). Early PRASE development work found a facilitated discussion at the patient's bedside to be the most successful in terms of volume, accuracy of patient reports, and “richness” of the data.^1^, ^6^ Although a facilitated discussion may introduce the potential of bias, offering participation in PRASE in this way aims to represent the best balance of richness of data, inclusivity in participation, and reduction of error in data collected.

**Figure 2:**
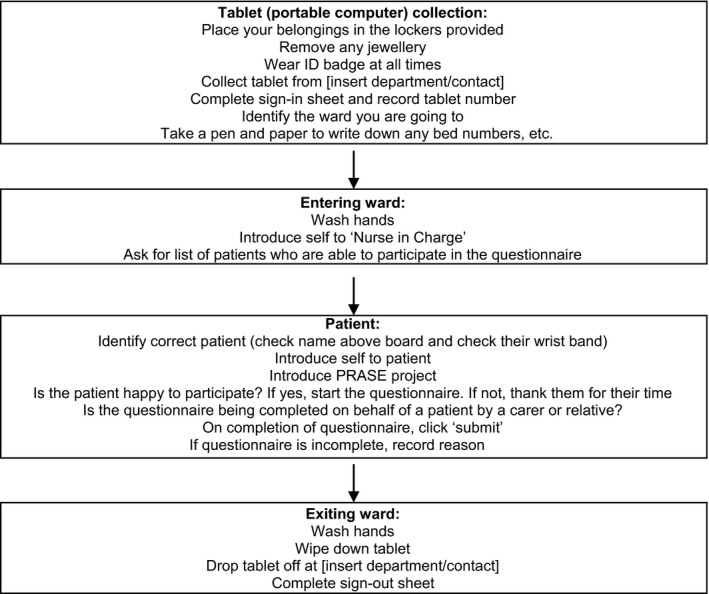
PRASE volunteer ward visit procedure

A common challenge following the development of evidence‐based interventions is how to spread innovation in practice.^10^ The PRASE intervention does not prescribe who should collect the patient feedback. However, to provide a sustainable mechanism beyond the research studies in which the intervention was developed and tested, an improvement project is exploring the potential for hospital volunteers to collect the patient feedback. The project is implementing the PRASE intervention in acute care settings. Figure 2 describes the procedure for PRASE volunteers visiting wards to collect patient feedback.

With healthcare organizations globally functioning with strained resources,^11^, ^12^, ^13^ and in the UK the National Health Service (NHS) under ever increasing pressure,^14^ paid ward staff have little or no spare capacity to undertake this role. Furthermore, there is evidence to suggest that patients prefer to report safety concerns to an independent party rather than through established, staff‐led reporting systems.^15^ Therefore, the rationale for the use of hospital volunteers stems from the need for a method of capturing the patient perspective of safety which is viewed as “independent” from the patient perspective which is not overly resource intensive.

Studies have shown that volunteers can provide cost‐effective support in resource constrained healthcare settings.^16^, ^17^ Research from the USA indicates that volunteers can offer significant cost savings to hospitals.^18^ Further, evidence is building that demonstrates the positive impact healthcare volunteers can have on patient experience and quality of care.^18^, ^19^, ^20^, ^21^

Recent findings from the first national survey focussing on the scale and value of volunteering in NHS acute hospitals highlighted that the average number of volunteers in an acute trust is 471. Administrative support (e.g general administration and administering patient surveys) was amongst the top five roles volunteers are engaged in.^22^ Indeed, the nature of the role of volunteers in healthcare is evolving, and volunteers are increasingly being called upon to facilitate the collection of patient feedback.^23^ However, there is currently little empirical evidence regarding the feasibility and acceptability of volunteers performing this role from an organizational perspective as well as from the perspective of the volunteers themselves. Therefore, we aim to address this gap within the context of PRASE.

This study explores the opportunities and challenges of implementing the PRASE intervention in collaboration with hospital volunteers within acute healthcare services. In this study, we present the results of a formative evaluation which spanned the pilot phase of the project. We explored the perceptions of key stakeholders (hospital volunteers, voluntary services, patient experience staff and ward staff) whose views we felt it particularly important to capture in the early phase of implementation. We intended to generate learning to contribute to the development of PRASE hospital volunteer recruitment, training, management and retention over the longer term. Involving hospital volunteers in the PRASE intervention is innovative; therefore, we know little about how being involved in this initiative impacts on the hospital volunteers over time. We anticipated that engaging voluntary services and patient experience teams would be crucial for the successful implementation of PRASE with hospital volunteers. Therefore, to embed PRASE in hospital routines in a sustainable way, we needed to understand the context around the PRASE intervention with hospital volunteers from the perspective of these staff. Finally, we were also interested in the implementation of PRASE with hospital volunteers from the perspective of ward staff, particularly their views on hospital volunteers collecting PRASE feedback. Furthermore, as ward staff are integral to the “active ingredient” hypothesized to facilitate service improvement within the PRASE intervention (the action planning process), it was essential their perceptions and experiences were explored in the pilot phase.

### 1.1 Research questions

Experiences of key stakeholders

1. What are the expectations, concerns and on‐going experiences of hospital volunteers involved in the PRASE intervention?
2. What are the expectations, concerns and experiences (e.g perceived barriers and levers) of voluntary services, patient experience and ward staff involved in the PRASE intervention with hospital volunteers?

Views on pilot implementation

- What are the key stakeholders’ views on the implementation of the PRASE

## 2 METHOD

### 2.1 Evaluation objectives and approach

There are many uses of formative evaluation in the context of implementation research.^24^ The main purpose of our formative evaluation was to address questions around feasibility and acceptability, and we aimed to provide the project implementation team with timely information to augment their learning prior to scale‐up and spread. A summative evaluation followed the formative work presented in this article. The summative evaluation phase focussed on whether the PRASE intervention with hospital volunteers resulted in improvements in quality and safety outcomes, as well as experiences of the key stakeholders over time.

Our evaluation approach was informed by recent publications which provide guidance on the evaluation of quality improvement work.^25^, ^26^, ^27^, ^28^ This literature posits a number of key principles that guided our evaluation approach: (i) flexibility and the need to be responsive to changes in implementation, (ii) iteratively revisiting the evaluation approach, and, (iii) the need to have an explicit programme theory, and a shared understanding of this amongst the wider project team. At the outset of the project, an earlier version of the PRASE intervention programme theory^6^ was adapted as a result of on‐going discussions between the wider project team. These adaptations aimed to reflect the addition of (i) hospital volunteer recruitment, training, management and retention, and, (ii) staff engagement to embed the PRASE intervention with hospital volunteers into routine ward activities. The programme theory delineated the hypothesized processes through which we expect the programme activities to result in both proximal and distal outcomes, with explicit reference to moderating factors known to affect these processes. We continually revised the programme theory throughout the pilot phase as a result of implementation learning and formative evaluation findings. We present a version of the programme theory which was agreed between the wider project team at the end of the pilot phase in the results section of this article. Being explicit about the programme theory allowed us to pinpoint the key stakeholders whose views it was important we capture within the formative evaluation.

### 2.2 Patient involvement in the design and conduct of the study

Patient representatives were involved throughout the development of the PRASE intervention, and along with clinical staff, helped to codesign the measurement tools.^29^ This project involved patient representatives in a number of ways. First, the research aim initially arose from discussions about the sustainability of the PRASE intervention with patients and healthcare professionals. Additionally, a patient representative was part of the initial application for funding, as well as invited to attend all steering group meetings. Their role was to provide input into both the implementation and evaluation components of the project, bring their experience as an active hospital volunteer, and to be involved in wider dissemination of the project progress.

### 2.3 Design

A multiple methods qualitative approach was adopted including focus groups with hospital volunteers and interviews with voluntary services, patient experience and ward staff which spanned the pilot phase of the project. We held multiple focus groups with hospital volunteers to account for turnover. The focus groups and interviews followed a semi‐structured format, and were mainly focussed around the participants’ early experiences of their involvement in PRASE and their views on implementation (e.g barriers and levers) with specific questions varying for each participant group. Follow‐up hospital volunteer focus groups revisited discussions from earlier focus groups where appropriate.

### 2.4 Setting and sample

Participants were recruited from two acute NHS trusts in the UK between July 2014 and November 2015. We conducted five focus groups with hospital volunteers (n=15), interviews with voluntary services and patient experience staff (n=3), and ward staff (n=4). Information pertaining to the characteristics of the participants is given in Table 1, 1.

**Table 1:** Characteristics of the participants

Hospital volunteer focus groups (FGs)	Duration	Sex	Age, mean (SD)
FG1	77 min	Female n=3; Male n=2	65.40 (8.20)
FG2	61 min	Female n=1; Male n=2	70.67 (0.58)
FG3	50 min	Female n=1; Male n=1	66.00 (4.24)
FG4	64 min	Female n=4; Male n=1	58.80 (21.38)
FG5	58 min	Female n=3	63.00 (4.36)
**Ward staff interviews**	**Duration**	**Job title**	
Interview 1	44 min	Consultant	
Interview 2	30 min	Senior Sister	
Interview 3	42 min	Consultant	
Interview 4	63 min	Consultant	
**Voluntary services/patient experience staff interviews**	**Duration**		
Interview 1	48 min		
Interview 2	47 min		
Interview 3	35 min		

### 2.5 Data analysis

The focus groups and interviews were digitally recorded and transcribed verbatim. We analysed the data taking a framework approach,^30^ and we used NVivo 10 Software for data management. The framework was developed iteratively by two researchers (GL and JOH), allowing for both a priori and emergent concepts and themes. Sections of the data were coded within corresponding themes (indexing) by one researcher (GL), and selected transcripts (n=3) were coded independently by the second researcher (JOH), with disagreements resolved through discussion. Within the framework, some concepts and themes related more so to the hospital volunteer focus groups and some more so to the staff interviews. Therefore, we present key themes for these stakeholder groups separately.

### 2.6 Ethics and governance

The appropriate governance approvals were sought for each research site, and ethical approval was granted by the University of Bradford, Humanities, Social and Health Sciences Research Ethics Panel (Hospital volunteer focus groups, ref: EC1578, May 2014; Voluntary services/patient experience staff interviews, ref: E440, April 2015; Ward staff interviews, ref: E463, July 2015).

### 2.7 Procedure

Hospital volunteers were invited to take part in the focus groups by a member of the local project implementation team. Invitations and participant information sheets which explained the purpose of the evaluation and the focus groups were distributed in advance, and dates for the focus groups were organized on behalf of the evaluation team. At one of the sites, three volunteers were involved from the very early stages of the project and therefore participated in two focus groups, which allowed us to follow up on their experiences. Staff were approached directly by an evaluation researcher and invited to participate in the interviews, and participant information sheets were distributed in advance of the interviews. Written informed consent was obtained from all participants. One of the main aims of the formative evaluation was to feed back early evaluation findings (within two weeks of data collection) to the project implementation team, enabling learning to be applied to the on‐going implementation of PRASE in a timely way. Given the small numbers of staff involved, to protect confidentiality, these participants were shown the evaluation findings we intended to share with the project implementation team in advance. Staff were given the opportunity to delete information and make amendments^2^.

## 3 RESULTS

We present key themes from the volunteer focus groups and staff interviews separately. Descriptions of each theme are provided, as well as illustrative excerpts. In addition, we present the participants’ views on implementation. We also reflect on the impact of evaluation activities on implementation and provide examples of how the formative evaluation findings informed changes to project implementation, and finally, how these findings relate to programme theory development.

### 3.1 The experience of hospital volunteers

Pertaining to the first research question “What are the expectations, concerns and on‐going experiences of hospital volunteers involved in the PRASE intervention?” three themes were identified from the hospital volunteer focus groups: (i) Hospital volunteers as collectors of patient feedback and utility of PRASE; (ii) Supporting hospital volunteers; and (iii) Skills, knowledge and experience. Reflections on pilot implementation (research question three) are incorporated throughout.

#### 3.1.1 Hospital volunteers as collectors of patient feedback and utility of PRASE

The hospital volunteers described the collection of patient feedback as essential in order for health services to improve. The belief that the PRASE intervention has the potential to result in valuable change to services was evident. There was an overwhelming sense that the volunteers bought into the philosophy of the PRASE intervention as a way to support service improvement, and acknowledged the potential benefits the PRASE volunteer role might bring to patients more immediately. Despite this enthusiasm, the volunteers discussed the context such service improvement interventions sit within, in particular the current pressures on the NHS in terms of staffing and resources. They reflected on how this “context” might bring about challenges for the PRASE intervention with volunteers; for example, one volunteer spoke about staff finding the time to provide the volunteers with the list of patients they can approach. The volunteers voiced pragmatic and ethical considerations around the implementation of PRASE. These included the procedures for identifying patients to approach and the need for this to be inclusive, particularly for non‐English speakers and patients who may not be able to give feedback themselves, as well as ensuring privacy at the bedside. There was much discussion of the two PRASE measurement tools (PMOS and PIRT). In these early stages of the project, the version of PMOS being used was the original 44‐item questionnaire. The volunteers believed that in its current format the questionnaire was too time consuming for patients to complete, and described its length as impacting on the conversation and rapport they were able to build with patients. Nevertheless, they did see the value in the PRASE measurement tools collecting in‐depth quantitative and qualitative information about the patient perspective of safety. See Box 1 for illustrative excerpts.

Box 1 **Hospital volunteer focus group excerpts reflective of the theme “Hospital volunteers as collectors of patient feedback and utility of PRASE”**

1 “…I think its got to be win, win for the hospital really and the staff on the wards because they're getting feedback about their good practice which is important and they're getting information about what could be improved…”

**Hospital volunteer 10, Female**

*“*The thing is though there's not enough staff on the wards in the first place so the ones that are there they're just overrun with everything and then all of sudden we turn up…”

**Hospital volunteer 3, Male**

#### 3.1.2 Supporting hospital volunteers

The volunteers described how the support mechanisms in place would determine their continued involvement, and that adequate support would be essential for PRASE volunteer retention. First, given the more in‐depth nature of the PRASE volunteering role, the need for the training to be fit for purpose was emphasized. As PRASE feedback is collected via a handheld tablet computer, many of the volunteers’ early views on training focussed on the technological side of the role and the importance of sufficient training and support around this. Consequently, in the initial focus groups the main anxieties for the volunteers were around using the technology. It was acknowledged that once they had more experience completing the questionnaires with patients their confidence would increase. In the early stages of the project, the volunteers felt they had not had sufficient training using the technology; they described not being given enough time to learn and go through things at an easy pace. The volunteers viewed their early involvement as “rushed” which may have impacted on their engagement. However, there was recognition that many of these issues were to be expected in a pilot scheme.

A handover/debrief after a patient recruitment session, and clear structures for emotional support were viewed as paramount, as well as guidance on when it is appropriate and necessary for the volunteers to escalate clinically significant information and who to escalate this to. On‐going group sessions with other PRASE volunteers were also advocated to facilitate peer support and shared learning. In addition to initial and further training when necessary, the volunteers were keen to receive feedback on their performance, as well as feedback on the actions wards have taken as a result of the feedback collected. There was a sense that their motivation to remain involved would decline if over a longer period of time the feedback had not being used to target improvements to services, or if the volunteers’ own motivations for participating were not being fulfilled. For both volunteer feedback on performance and fulfilling expectations, we suggest a certain amount of tailoring may be required. This is due to the volunteer's describing different motivations for participating (e.g an experience as a patient, a previous job, a need to be doing something, a desire to “give back”) and differing expectations in terms of the level of feedback on performance they would like to receive. The importance of role clarity was emphasized, both in terms of the volunteers’ role boundaries and expectations, but also the need for patients and staff to be aware of PRASE to facilitate the volunteers’ communication with these groups. See Box 2 for illustrative excerpts.

Box 2 **Hospital volunteer focus group excerpts reflective of the theme “Supporting hospital volunteers”**

1 *“*It would be nice for us to have feedback as to how we've been and to know this has made a difference or if it's not working…”

**Hospital volunteer 1, Female**

[Referring to group sessions]”*…*you could talk about your different experiences on the wards and different things that have cropped up… and you can say well how did you cope with that one, you know, how did you get round that one…”

**Hospital volunteer 2, Male**

*“*And I think my own feeling, this is well due, something like this because having been in hospital and by and large the care has been perfect, the care has always been good but the procedures might not be so good and it seems uncharitable to complain…”

**Hospital volunteer 9, Female**

#### 3.1.3 Skills, knowledge and experience

The volunteers acknowledged the diverse skill set and knowledge required for the PRASE role, some of which are also required for other hospital volunteer roles. Emphasis was placed on the ability to balance the emotional aspects of the role and communicate effectively with both staff and patients, and an understanding of patient safety. In addition to skills around the use of the technology, being able to navigate a ward environment and maintain a neutral and diplomatic stance when collecting patient feedback were also recognized. Suitability for the PRASE role in terms of possessing the right skills and knowledge seemed to be facilitated by the volunteers’ previous experience, both in terms of volunteering in a hospital setting and previous employment, for example, in a healthcare/NHS setting. The vital role of PRASE training and support in ensuring the volunteers develop the skills and knowledge required was evident. See Box 3 for illustrative excerpts.

Box 3 **Hospital volunteer focus group excerpts reflective of the theme “Skills, knowledge and experience”**

1 *“…*interpersonal skills, you know, to actually relate to someone at various levels on it. It would be nice to have different languages as well like, you know, being able to build a quick rapport isn't it, you know, and listening, active listening as well…”

**Hospital volunteer 7, Male**

*“…*being impartial because, yes I understand that we're not there to influence the patient in any way shape or form. I think that's a difficult thing but, you know, you've to deal with it.”

**Hospital volunteer 6, Female**

### 3.2 The experience of staff

Research question two focussed on the expectations, concerns and experiences of voluntary services, patient experience and ward staff involved in the PRASE intervention with hospital volunteers. Three themes emerged from the staff interviews: (i) Involving volunteers in PRASE; (ii) Moving from data to action; and (iii) Sustainability. Reflections on pilot implementation (research question three) are incorporated throughout.

#### 3.2.1 Involving volunteers in PRASE

Staff were overwhelmingly supportive of the implementation of PRASE with hospital volunteers. Given their independent standing, staff viewed volunteers as well placed to collect the feedback. However, even as part of a pilot scheme the infrastructure needed for the implementation of PRASE with volunteers was viewed as substantial, the management and organization of which over the longer term would require dedicated capacity. In the early stages of the project, volunteers were recruited from existing hospital volunteer cohorts, and subsequently a person specification was developed for the PRASE volunteer role, which was advertised both internally and externally. Staff emphasized the need for this targeted recruitment to ensure volunteers with the right skills are identified for the role. Staff felt the volunteer training was fit for purpose, but again time consuming when factoring in volunteer retention, as recruitment and training would need to be part of a rolling programme. Given the in‐depth nature of the role, in addition to the initial induction and PRASE training which was usually classroom based, individual ward training where the volunteers visit the ward with staff (voluntary services/patient experience) was necessary. In terms of supporting the volunteers on an on‐going basis, that is what was required, how this was provided and by whom, this differed depending on the voluntary services set up at each site. For example, at one site this was led by voluntary services, whereas at the second site this was viewed as a collaborative effort between the patient experience team and the ward/department a volunteer was assigned to. Despite these differences, there was agreement about the type of support the volunteers require, which included the following: emotional support; handover after patient recruitment session; clear procedures for the escalation of concerns; feedback on performance; and peer support. Linked to this, the importance of fulfilling the needs of individual volunteers was recognized to increase the likelihood of volunteer retention.

In addition to patient variation, there was an acknowledgement that there would be variation in how the volunteers collect the feedback and the supporting infrastructures in place which may have implications for data quality. This was mainly raised as an issue by ward staff who would be using the feedback to support improvements, and it was suggested that the provision of appropriate training and support may alleviate this potential variation. There was much discussion of the logistical and practical considerations surrounding PRASE with volunteers, not least the organization of volunteers and how this fits within existing workloads, but also the unpredictability of the ward environment and patient availability. Regarding staff views of pilot implementation, many staff found the project management structure to be somewhat unclear across the sites, but again there was recognition that this was to be expected in a pilot scheme. See Box 4 for illustrative excerpts.

Box 4 **Staff interview excerpts reflective of the theme “Involving volunteers in PRASE”**

1 *“*I think in principle it is a good idea. It is in keeping with the national impetus. I think that Francis mentioned use of volunteers. It's been topic of…. I think the problem is going to be the logistics, and certainly if this was to sort of to be used throughout the hospital, the machinery involved in getting people trained retrained emm telling where they are going supporting them is absolutely massive…”

**Ward staff**

*“…*in volunteer services there's only [name] and their role is to recruit but obviously we've got 300 hundred volunteers in the hospital, it's not possible for [name] to nurture people in the way that, it's up to teams who want the volunteers to nurture them themselves so I would see that as our role because we're patient experience. I would see it as a dual role with ourselves and whichever ward they were allocated to…”

**Patient experience staff**

#### 3.2.2 Moving from data to action

Although we were principally interested in the added dimension of involving hospital volunteers in PRASE, many of the discussions with ward staff focussed on their views of the intervention more generally. PRASE from the perspective of ward staff has been explored comprehensively in the embedded qualitative process evaluation^8^ within the randomized controlled trial of the intervention,^5^ and many of our findings support some of the conclusions of this previous work. Staff viewed capturing patient feedback for service improvement as essential, and saw PRASE as a meaningful way of achieving this. However, some potential limitations were also discussed. For example, if over the longer term staff continue to receive feedback they are not able to put into action—for example because some issues are difficult to address at the ward level—their engagement in the process may decline. So although staff liked the fact the PRASE measurement tools collected more detail and context, there was a sense that the tools need to be fit for purpose if the aim of data collection is to lead to actionable change. Linked to this, the amount of information within a feedback report considered at a meeting needs to be manageable. Staff also described barriers around the logistics of the action planning element of the intervention, in terms of getting the right staff together and finding time for these meetings. These challenges which relate to the intervention more generally, that is using the feedback to support actionable change, could have implications for the implementation of PRASE with volunteers, who may lose motivation to remain involved if they perceive the feedback collected has not led to improvements to services. The role of facilitation was viewed as integral to this and staff described the potential benefit of multiple ward action planning meetings being facilitated by the same person, as this person would then have an overview of key issues across wards and would be well positioned to share solutions. Staff were supportive of the suggestion that volunteers could be involved in the action planning meetings as long as the rationale behind this was clearly articulated, they saw the potential benefit of the volunteers further expanding on the data collected at these meetings. Over the longer term, staff felt ward engagement and ownership of the data would be essential levers to PRASE being successfully embedded into routine practice. See Box 5 for illustrative excerpts.

Box 5 **Staff interview excerpts reflective of the theme “Moving from data to action”**

1 *“…*but there were some things that seemed really kind of obvious when you see it written down that yeah why aren't we doing that for example and also could readily be sorted with not a lot of resource and no financial kind of input and perhaps just changes in practice kind of thing…”

**Ward staff**

[Referring to action planning meetings] *“…*So I think a good facilitator will help to eat the elephant one bite at the time. Which is needed and get to the real issues rather than be tied up with the periphery of it. Stuff that isn't well, it is important but only if it is indicating broader concern about you know communication for instance. It is not the individual exchanges that are crucial in that case. So yes, I think they need to be facilitated…”

**Ward staff**

#### 3.2.3 Sustainability

Discussions with staff often focussed on what PRASE with volunteers might look like in the future, and how PRASE could be embedded in a sustainable way. Staff voiced many reservations about sustainability. In terms of scale‐up and spread, staff felt buy‐in from higher up the organization would be essential if PRASE with volunteers was to become more widespread. From the voluntary services and patient experience staff perspective, if PRASE were scaled up, dedicated staff capacity would be needed to support the infrastructure (i.e volunteer recruitment, training and on‐going support). For ward staff, the challenge of getting staff together for the action planning meetings spoke to a broader issue, that is the lack of dedicated time for staff to “think about safety.” Staff also described the need for new initiatives to consider the current NHS context and existing initiatives. Relatedly, staff voiced that how PRASE feedback sits with other sources of patient feedback needs to be considered, and stressed the need for initiatives with similar aims to “tie together.” Finally, although staff were supportive of PRASE and its potential benefits, concerns were raised around the evidence base in terms of improving patient safety outcomes and demonstrating cost‐effectiveness. See Box 6 for illustrative excerpts.

Box 6 **Staff interview excerpts reflective of the theme “Sustainability”**

1 *“…*If you were doing it Trust wide, you'd definitely need another person. At the moment we can just about absorb it…”

**Voluntary services staff**

*“…*Whether you could ever get the volume of volunteers for a Trust wide thing, and how do you deal with people, you know how long do you do it for, we've not really got into it, do you do it for one cycle, do you do it for a year, do you do it for three years. There is fatigue….you know I get the sense that we will have to keep rotating them, and that means you have to keep bringing new sets in. So you can imagine, it's like an industry, it is just like generating a new industry.”

**Ward staff**

### 3.3 Impact of evaluation activities and early findings on implementation

In undertaking focus groups with volunteers and interviews with staff, it became clear that there was a sense of uncertainty, for example, about the specifics of the PRASE intervention and project timelines. Therefore, participants valued the opportunity to share their experiences, get peer support (volunteers), and ask questions that they had about their wider understanding of the intervention. Whilst supporting the volunteers and staff is of course a useful activity, and we were pleased that they felt that participation was beneficial for them, it did highlight to us the issue of reflexivity in our evaluation activities. In terms of how the formative evaluation findings were incorporated into project implementation, to give a few examples, the findings contributed to the volunteer retention plan (i.e the inclusion of regular volunteer group “catch‐up” sessions). Additionally, the findings led to the PMOS questionnaire being shortened to 30 items by the research team who developed the original questionnaire, and the recommendation of hospital volunteer involvement in ward action planning meetings.

### 3.4 Programme theory development

Figure S1 provides a diagrammatic representation of the programme theory (logic model) for the implementation of the PRASE intervention with hospital volunteers. This version of the programme theory was agreed between the wider project team at the end of the pilot phase, and was informed by implementation learning and the formative evaluation findings. Example modifications to the programme theory included recognizing the engagement of, and support from clinical leads and ward staff as key moderating factors, and the addition of “awareness and buy‐in from specialty areas” as a proximal outcome.

## 4 DISCUSSION

### 4.1 Description of main findings in relation to existing literature

Hospital volunteers saw the value of their role in the PRASE intervention and the potential benefits for patients and for service improvement, but they also recognized that how the PRASE intervention sits in terms of the current NHS context (e.g staffing issues) needs consideration. The volunteers stressed the importance of ensuring PRASE was inclusive to as many patients as possible, and the need for the PRASE measurement tools to be fit for purpose. Referring to their involvement over the longer term, the volunteers felt the training and support mechanisms (e.g handover/debrief, group sessions, feedback on performance) would be influential, and this tied in with the different motivations volunteers had for their involvement, such as a desire to “give back” in a way that was also rewarding and a “need to be doing something.” When we look to the wider literature around hospital volunteering, these findings are in line with research that highlights the importance of organizations understanding volunteers’ motivations and meeting their expectations for retention.^31^, ^32^

As some of the elements of the PRASE volunteer role are different to traditional hospital volunteer roles, the volunteers described a diverse skill set which they believed to be important, for example communicating effectively with staff and patients, balancing the emotional aspects of the role and remaining neutral. Access to adequate support around the emotional aspects of the PRASE volunteer role seemed to be crucial. Indeed, the need for volunteer managers to be aware of how factors such as stress and sadness may affect volunteers in a particular role, and to provide the necessary support has been acknowledged in the wider literature.^33^

Reflecting on the main findings from the voluntary services and ward staff perspective, overwhelmingly staff were enthusiastic about the potential for PRASE to support meaningful service improvement. They were unanimous in their belief that volunteers were well placed to collect feedback from patients given their independent standing. The importance of targeted recruitment was emphasized, and the need to fulfil the needs of individual volunteers to ensure retention. Concerns were raised around the resource and infrastructure required, with staff voicing reservations about sustainability over the longer term. Staff felt the management and organization of PRASE with hospital volunteers would require dedicated capacity from voluntary services and/or patient experience teams, and that buy‐in from higher up an organization would be essential if PRASE delivered in collaboration with hospital volunteers was to become more widespread. Despite staff reservations around sustainability and the resource and infrastructure required for PRASE with volunteers, previous research has suggested that the economic benefits of hospital volunteers offset the costs, even when the costs associated with screening, training, supervising and other administrative costs are factored in.^20^ Although this previous research is promising, it did not specifically focus on volunteers facilitating patient feedback collection and a cost–benefit analysis of the implementation of PRASE with volunteers in terms of voluntary services and patient experience team resource was outside the scope of this formative evaluation.

Ward staff appreciated that the PRASE measurement tools allowed for the collection of more detailed feedback, but also described how these tools need to be fit for purpose; for instance, engagement may decline if over a longer period of time staff continue to receive patient feedback they feel they are not able to put into action. The facilitation of the action planning meetings was said to be vital, which mirrors previous research highlighting the need for the facilitation of staff groups to interpret patient experience feedback, to aid staff to make sense of the feedback for appropriate service improvements.^34^ The difficulties of getting the right staff together for these meetings and “finding time to think about safety” were emphasized, and there was a sense that when introducing a new intervention, consideration needs to be given to how the intervention ties in with other initiatives, and other sources of patient feedback available to wards. These findings speak to on‐going debates around the use and usefulness of patient feedback for service improvement, in particular the capacity for staff to make sense of the different forms of patient feedback.^35^, ^36^, ^37^ Finally, staff were supportive of the potential for involving hospital volunteers in the action planning meetings, recognizing the benefits this different perspective could bring. Policymakers, managers and practitioners advocate the value of involving patients in planning and improving health services, but there is limited research around the proactive involvement of patients in the “planning” of ways to improve services.^38^ The philosophy underpinning PRASE encourages a continuous cycle of measurement, feedback and action planning. The implementation of PRASE with hospital volunteers offers a unique opportunity to address this in some way, by involving the volunteers who collect the patient feedback in the “planning” for service improvement.

### 4.2 Implications

One of the main aims of the formative evaluation was to provide the implementation team with timely information which could be incorporated into project implementation. We achieved this aim, with our findings directly contributing to the volunteer retention plan (i.e the inclusion of regular volunteer group “catch‐up” sessions), the shortening of the PMOS questionnaire, and the recommendation of hospital volunteer involvement in ward action planning meetings. The formative evaluation findings informed the refinement of the programme theory for PRASE delivered in collaboration with hospital volunteers which was taken forward to the roll‐out phase of the project. Regarding the use of hospital volunteers to facilitate the collection of patient feedback in health care more generally, which could be viewed as a departure from traditional hospital volunteering roles, our findings emphasize the need for targeted recruitment and adequate training and support, with particular support available for coping with the emotional consequences of collecting feedback directly from patients on a regular basis. However, we do of course recognize the more substantial nature of the PRASE volunteer role in comparison with other ways volunteers have been involved in patient feedback collection (i.e facilitating the collection of “Friends and Family Test”^39^ in the UK).

### 4.3 Limitations

This was a formative evaluation of an improvement initiative, meaning our research activities were limited to the organizations involved in the wider project. It is possible that this had implications in terms of generalizability, as the findings may be specific to the context of the two NHS trusts involved. However, given that our findings reflect issues from the wider literature on hospital volunteering, we are confident our findings are applicable more broadly. A further limitation of this formative evaluation is that the patient perspective was not within the scope of the study, in the sense that we did not explore patients’ views of the implementation of PRASE with hospital volunteers. We know from previous research that patients prefer to report safety concerns to an independent party rather than through established, staff‐led reporting systems,^15^ and there is evidence demonstrating the success of collecting patient‐reported safety concerns by facilitated discussion at the patient's bedside.^1^ However, we do not know if the volunteers are viewed as “independent” from the patient's perspective. Although the need to maintain a neutral and diplomatic stance was acknowledged, given the many aforementioned motivations for participating, it is essential for the volunteer training and on‐going support to reinforce the importance of impartiality and role boundaries to mitigate any concerns about perceived independent status of the volunteers. In the light of the above limitations, we recommended that future research explores patients’ views of hospital volunteers facilitating the collection of patient feedback, to understand whether this approach is feasible and acceptable to patients. We also advocate that research focusses on the impact of the PRASE intervention delivered in collaboration with hospital volunteers on quality and safety outcomes, in addition to cost‐benefit analyses.

### 4.4 Conclusion

This theory‐led qualitative formative evaluation suggests the PRASE intervention in collaboration with hospital volunteers is feasible and acceptable to key stakeholders, whilst recognizing barriers from the perspectives of these stakeholders which need to be addressed. The formative evaluation findings were incorporated into project implementation, and offer valuable insights into the use of hospital volunteers as facilitators of patient feedback collection more generally.

## COMPETING INTERESTS

None declared.

## AUTHORS’ CONTRIBUTIONS

All authors developed the design of the formative evaluation. GL conducted the data collection and analysis, with input from JOH. GL drafted the manuscript, and all authors provided comments and approved the final version.

## Notes

1 To protect the anonymity of the participants, we have not included the job titles of the voluntary services and patient experience staff.

2 One participant clarified the impact of PRASE on the voluntary services workload, and one participant expanded on a point they had made regarding senior input in PRASE ward action planning meetings. No information was deleted.
