# Supplementary material for: A qualitative formative evaluation of a patient‐centred patient safety intervention delivered in collaboration with hospital volunteers
Source: Health Expect. 2017 Jun 15;20(5):1143–53. doi: 10.1111/hex.12560 (PMC5600221; doi:10.1111/hex.12560)
Supplement: Supplementary file 1 [file HEX-20-1143-s001.doc]

Improved patient safety/quality performance

Improved safety culture

Patient-centred service reconfiguration

Collaborative, multi-disciplinary approach to service improvement

Distal outcomes

Proximal outcomes

Programme activities

Key moderating factors

Engagement of, and support from senior management, clinical leads and ward staff

Perceived credibility and usefulness of patient feedback

Knowledge and capability in action planning/quality improvement

Perceived control and self-efficacy to effect change

Multi-disciplinary approach to action planning and implementing change

Scope of proposed actions

Engagement of, and support from voluntary services and patient experience teams

Engagement of, and support from volunteers

Patient experience of safety measured

Information collated and fed back to wards

Feedback considered in Action Planning Group

Action Planning Group plan, implement and monitor changes

Volunteer recruitment, training, management and retention

*Figure S1. Logic model depicting programme theory for the PRASE intervention in partnership with hospital volunteers*
